# Pre-Hospital Dietary Intake Correlates with Muscle Mass at the Time of Fracture in Older Hip-Fractured Patients

**DOI:** 10.3389/fnagi.2014.00269

**Published:** 2014-11-19

**Authors:** Riccardo Calvani, Anna Maria Martone, Emanuele Marzetti, Graziano Onder, Giulia Savera, Maria Lorenzi, Elisabetta Serafini, Roberto Bernabei, Francesco Landi

**Affiliations:** ^1^Department of Geriatrics, Neurosciences and Orthopedics, Teaching Hospital “Agostino Gemelli”, Catholic University of the Sacred Heart School of Medicine, Rome, Italy

**Keywords:** sarcopenia, diet, recommended dietary allowance, leucine, disability, malnutrition, bioelectrical impedance analysis, muscle atrophy

## Abstract

**Background:** Failure to meet an adequate dietary intake is involved in the pathogenesis of sarcopenia and osteoporosis, which in turn increase the risk for falls and fractures, respectively. Older people with hip fracture are often protein-malnourished at hospitalization. Whether low protein-energy intake is associated with muscle atrophy in hip-fractured patients is presently unknown. This information is necessary for the development of novel strategies to manage this especially vulnerable patient population. The aim of this study was, therefore, to explore the relationship between dietary intake and muscle mass in older hip-fractured patients.

**Methods:** Analyses were conducted in hip-fractured elderly admitted to an orthopedic and trauma surgery ward (University Hospital). Muscle mass was estimated by bioelectrical impedance analysis within 24 h from admission. Dietary information was collected via 24-h dietary recall and nutrient intake calculated by a nutrition software.

**Results:** Among 62 hip-fractured patients (mean age 84.6 ± 7.6 years, 84% women), the average energy intake was 929.2 ± 170.3 Kcal day^−1^, with higher values reported by men (1.046.8 ± 231.4 Kcal day^−1^) relative to women (906.5 ± 148.3 Kcal day^−1^; *p* = 0.01). Absolute and normalized protein intake was 50.0 ± 13.5 g day^−1^ and 0.88 ± 0.27 g kg (body weight)^–1^ day^–1^, respectively, with no gender differences. A positive correlation was determined between total energy intake and muscle mass (*r* = 0.384; *p* = 0.003). Similarly, protein and leucine consumption was positively correlated with muscle mass (*r* = 0.367 and 0.311, respectively; *p* = 0.005 for both).

**Conclusion:** A low intake of calories, protein, and leucine is associated with reduced muscle mass in hip-fractured elderly. Given the relevance of sarcopenia as a risk factor for adverse outcomes in this patient population, our findings highlight the importance of a comprehensive dietary assessment for the detection of nutritional deficits predisposing to or aggravating muscle atrophy.

## Introduction

Hip fracture is a devastating event for elderly people, with over 25% per-year mortality and incomplete recovery of pre-fractural conditions in more than 50% of survivors (Maggi et al., 2010). Approximately 1.6 million older adults worldwide sustain a hip fracture annually (Hung et al., 2012). What is worse, due to the ongoing demographic transition, the incidence of hip fractures is projected to increase up to 2.6 million by 2025 and reach 4.5 million in 2050 (Cauley et al., 2014). This epidemiological figure has a dramatic impact from both healthcare and societal perspectives, given the enormous direct (e.g., acute in-hospital treatment, rehabilitation programs, and use of health services) and indirect costs (e.g., burden to families related to the patient’s acquired or worsened disability) associated with hip fracture and its consequences (Pike et al., 2010). These considerations call for the development of novel strategies to improve the survival and functional recovery of this vulnerable patient population.

Among the factors that may impact the clinical outcome of hip-fractured elderly, the age-related loss of muscle mass and function (sarcopenia) emerges as serious candidate for interventions. Indeed, declines in muscle mass and strength are associated with poor functional recovery following hip fracture repair (Visser et al., 2000; D’Adamo et al., 2014). Given the role of protein–energy malnutrition as a risk factor for the development of sarcopenia (Calvani et al., 2013; Landi et al., 2013a; Martone et al., 2013), a suboptimal nutritional status may mediate, at least partly, the association between sarcopenia and poor clinical outcomes in older hip-fractured patients.

Malnutrition is commonly found in older adults admitted to hospital with hip fracture (Murphy et al., 2000). Moreover, older hip-fractured patients who enter the hospital undernourished do not usually meet the recommended dietary allowance (RDA) for protein [0.8 g kg (body weight)^–1^ day^–1^] (Miller et al., 2006). This in the face that a high protein intake reduces the risk of perioperative complications (Milne et al., 2009; Botella-Carretero et al., 2010), improves bone mineral density (Schürch et al., 1998; Tengstrand et al., 2007), and shortens the rehabilitation time in this patient population (Avenell and Handoll, 2005). Nevertheless, a debate is ongoing as to whether the current RDA for protein is sufficient to prevent major adverse events in older adults, especially in frail, critically ill patients (Bauer et al., 2013).

Although one may expect a relationship exists that links low protein and energy ingestion, muscle atrophy, and poor clinical outcomes, no studies have yet assessed the association between dietary intake and muscle wasting in hip-fractured older patients. If such an association does exist, it would imply that a potentially amenable causative factor of sarcopenia and related consequences may be proposed as a therapeutic target in standard clinical practice. The present study was, therefore, undertaken to verify the association between dietary intake and muscle atrophy in a sample of hip-fractured elderly, with the aim of providing the foundation for future intervention studies.

## Materials and Methods

### Study sample

The study was performed between November 2012 and August 2013 among older adults admitted for hip fracture due to accidental fall to the Emergency Department (ED) of the Teaching Hospital “Agostino Gemelli,” Catholic University of the Sacred Heart (Rome, Italy). Exclusion criteria were age < 65 years, presence of peripheral edema, bone metastasis, cognitive impairment (Cognitive Performance Scale < 3), presence of pacemaker or implantable cardioverter defibrillator, and unwillingness to take part to the study. The study was approved by the Institutional Review Board of the Catholic University of the Sacred Heart, and all participants signed a written consent before enrollment.

### Data collection

Information pertaining to demographic, clinical, functional, and lifestyle characteristics were collected by attending physicians upon admission to the Orthopedic and Trauma Surgery ward using the interRAI Acute Care instrument (Gray et al., 2008). Due to practical difficulties in obtaining accurate weight and height measurements in our patient population, body mass index (BMI) was calculated based on self-reported weight and height. However, an excellent agreement has been shown in older adults between self-reported and measured anthropometric parameters (Ng et al., 2011).

### Assessment of muscle mass

Whole-body fat-free mass was measured by bioelectrical impedance analysis (BIA) using a Quantum/S Bioelectrical Body Composition Analyzer (Akern Srl, Florence, Italy) with an operating frequency of 50 kHz at 800 μA, as previously described (Marzetti et al., 2014). Measurements were taken within 24 h from ED admission adopting standard conditions (NIH Expert Panel, 1996), with the subject in a supine position and surface electrodes placed on wrist and ankle contralateral to the side of the fracture. Muscle mass was estimated using the equation developed by Janssen et al. (2000). The skeletal muscle index (SMI) was obtained dividing absolute muscle mass by squared height (kg m^−2^).

### Dietary assessment

Pre-hospital dietary information was collected within 24 h from admission to the Orthopedic and Trauma Surgery ward via a dietary interview on nutritional habits. The nutrient intake was estimated by 24-h dietary recall of the day before fracture (Buzzard, 1998). Collected data were elaborated using a nutrition software (MètaDieta, ME.TE.DA. LLC, San Benedetto del Tronto, Italy) to estimate the daily intake of macro- and micronutrients.

### Statistical analyses

All data are expressed as proportions (%) or mean ± standard deviation (SD). Differences between continuous variables were assessed by ANOVA comparisons or the Kruskal–Wallis test, as appropriate. Distributions of categorical variables were compared by the Fisher exact test. The Pearson’s correlation test was used to assess the strength of association between variables. All tests were two sided, with significance set at *p* < 0.05. All analyses were run using the SPSS software (version 18, SPSS Inc., Chicago, IL, USA).

## Results

The main characteristics of the study sample are shown in Table 1. The mean age of participants was 84.6 ± 7.6 years, with no differences between genders. Women were predominant (84%), which is coherent with epidemiological data showing a higher incidence of hip fracture in the female gender (Cauley et al., 2014). Men showed a trend toward higher muscle mass values relative to women (SMI: 10.00 ± 2.70 kg m^−2^ and 8.83 ± 1.73 kg m^−2^, respectively), but the difference did not reach the statistical significance (*p* = 0.08) (Table 1).

**Table 1:** **Characteristics of study participants according to gender**.

	Total (*n* = 62), *n* (%)	Men (*n* = 10), *n* (%)	Women (*n* = 52), *n* (%)	*p* value
Age (years) (mean ± SD)	84.6 ± 7.6	86.1 ± 4.7	84.4 ± 8.1	0.5
Pre-fracture ADL score (mean ± SD)^a^	1.4 ± 2.4	1.2 ± 2.2	0.8 ± 1.5	0.7
CPS score at admission (mean ± SD)^b^	0.8 ± 1.5	0.9 ± 1.6	0.8 ± 1.5	0.8
Number of diseases (mean ± SD)	4.8 ± 3.3	6.1 ± 2.9	4.6 ± 3.4	0.1
Number of medications (mean ± SD)	2.7 ± 1.7	4.1 ± 1.7	2.4 ± 1.6	0.03
BMI (mean ± SD)	22.0 ± 3.3	22.1 ± 2.8	23.2 ± 3.3	0.3
SMI (kg m^−2^) (mean ± SD)	9.02 ± 1.9	10.00 ± 2.70	8.83 ± 1.73	0.08
Pre-fracture daily energy intake (kcal) (mean ± SD)	929.2 ± 170.3	1046.8 ± 231.4	906.5 ± 148.3	0.01
Pre-fracture daily protein intake (absolute) (g) (mean ± SD)	50.0 ± 13.5	55.3 ± 16.6	50.8 ± 14.0	0.2
Pre-fracture daily protein intake (normalized) (g kg^–1^) (mean ± SD)	0.88 ± 0.27	0.86 ± 0.31	0.88 ± 0.26	0.9
Pre-fracture daily leucine intake (g) (mean ± SD)	3.97 ± 1.13	4.40 ± 1.39	3.89 ± 1.07	0.1

*ADL: activities of daily living; BMI: body mass index; CPS: Cognitive Performance Scale; SMI: skeletal muscle index*.

*^a^ADL: 0 (no impairment), 7 (severe impairment)*.

*^b^CPS: 0 (no impairment), 6 (severe impairment)*.

**Figure 1:**
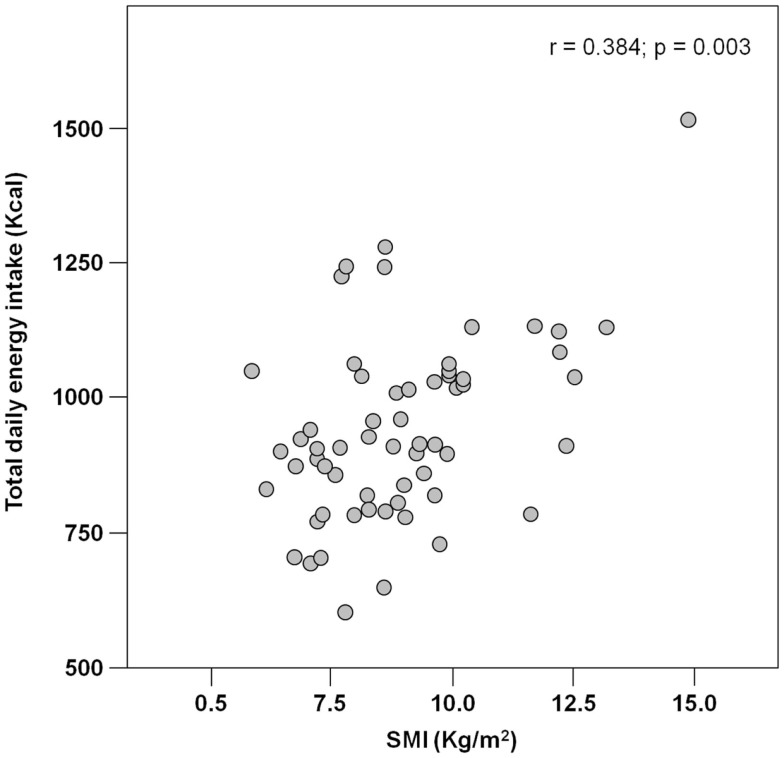
**Scatter plot of total daily energy intake and skeletal muscle index (SMI; *n* = 62)**.

**Figure 2:**
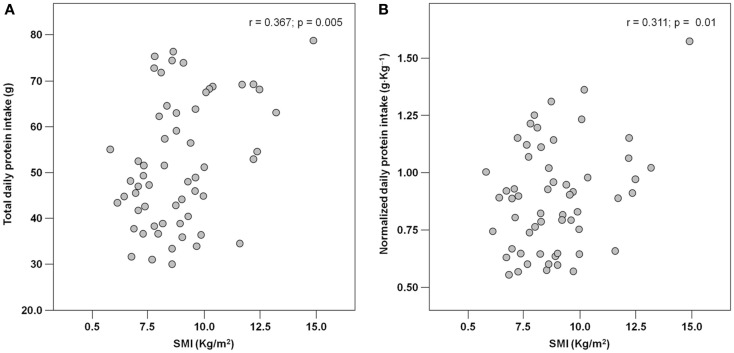
**Scatter plot of total (A) and normalized (B) protein intake and skeletal muscle index (SMI; *n* = 62)**.

**Figure 3:**
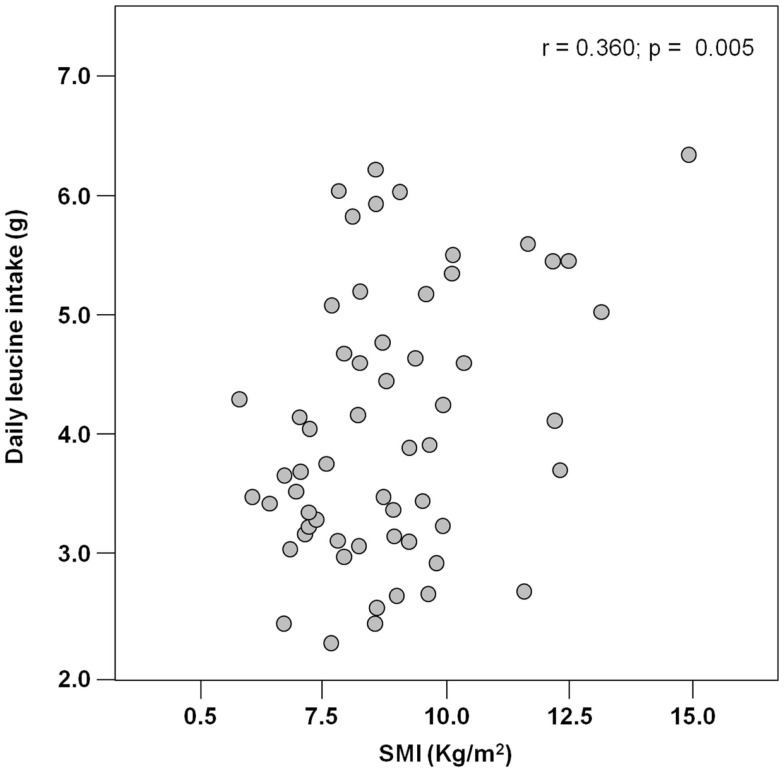
**Scatter plot of daily leucine intake and skeletal muscle index (SMI;*n* = 62)**.

The mean pre-hospital energy intake was 929.2 ± 170.3 kcal day^−1^, with a significantly higher energy consumption reported by men (1046.8 ± 231.4 vs. 906.5 ± 148.3 kcal day^−1^; *p* = 0.01; Table 1). Pre-hospital protein intake was 50.0 ± 13.5 g day^−1^, corresponding to 0.88 ± 0.27 g kg (body weight)^–1^ day^–1^, with no differences between genders (Table 1). Remarkably, more than 75% of participants reported protein consumption below 1.0 g kg^–1^ day^–1^, which represents the minimum intake currently recommended for older people (Bauer et al., 2013; Deutz et al., 2014). Finally, the average leucine consumption was 3.97 ± 1.13 g day^–1^, with similar values in men and women (Table 1). A positive correlation was determined between SMI and total daily energy intake (*r* = 0.384; *p* = 0.003; Figure 1). Positive, significant correlations were also found between SMI and both absolute (*r* = 0.367, *p* = 0.005; Figure 2A) and normalized daily protein intake (*r* = 0.311, *p* = 0.01; Figure 2B), as well as daily leucine consumption (*r* = 0.360, *p* = 0.005; Figure 3).

## Discussion

Hip fracture is a dramatic event for older adults due to its detrimental consequences on the individual health status and quality of life. Considerable efforts have, therefore, been directed toward the development of interventions aimed at reducing the incidence of new fractures and improving health outcomes when the fracture has occurred (Hung et al., 2012; Pioli et al., 2014). Over the years, a shift of paradigm has emerged such that hip fracture is no longer considered as a condition limited to the bone. Rather, it represents a complex “geriatric syndrome” that affects the whole organism (Pioli et al., 2014). As such, it is becoming increasingly clear that the management of this condition requires a comprehensive, multidisciplinary approach that goes beyond fracture repair and osteoporosis treatment (Hung et al., 2012; Pioli et al., 2014). Our results should be considered against this complex clinical and pathophysiological backdrop.

Previous investigations have shown that older people with hip fracture are often energy and protein malnourished at the time of fracture (Lumbers et al., 2001) and that a high prevalence of sarcopenia is observed in this patient population (Di Monaco et al., 2011, 2012). The finding from the present study that these two phenomena are correlated with each other extends our knowledge on these frail patients and adds interesting cues on hip fracture management. Indeed, muscle wasting is a powerful risk factor for adverse outcomes among older adults hospitalized with acute conditions. For instance, recent data from members of our group have shown that among 770 older patients admitted to acute care units, participants with sarcopenia experienced a threefold higher in-hospital mortality as compared with non-sarcopenic patients (Vetrano et al., 2014). This association remained significant after adjustment for a number of potential confounders, including among others cancer, cardiovascular disease, chronic obstructive pulmonary disease, dementia, chronic kidney disease, and pre-hospital disability. Furthermore, reduced muscle mass and strength were found to predict poor mobility recovery following hip fracture repair (Visser et al., 2000; Di Monaco et al., 2007).

On the other hand, it is well established that an adequate intake of dietary protein is required for the preservation of lean body mass in late life (Calvani et al., 2013). For instance, in the Health, Aging, and Body Composition Study, older adults in the highest quintile of protein consumption lost nearly 40% less appendicular lean mass than did those in the lowest quintile over 3 years of follow-up, after adjustment for potential confounders (Houston et al., 2008). Notably, protein supplementation is *per se* sufficient at increasing muscle mass in older hospitalized patients (Bos et al., 2001). This effect may explain, at least partly, the improvement in clinical outcomes observed in hip-fractured elderly undergoing perioperative protein supplementation (Bauer et al., 2013).

A central aspect to consider is that a higher dietary protein ingestion is necessary for the promotion of muscle health in older persons relative to young adults (Bauer et al., 2013). In light of this evidence, the average protein intake recorded in our study sample (0.88 g kg^–1^ day^–1^), albeit slightly above the RDA for protein, may not still be sufficient to sustain optimal muscle protein synthesis. Notably, over 75% of participants did not reach the protein intake currently recommended for older adults (1.0 g kg^–1^ day^–1^), and only 8% reported a protein consumption between 1.2 and 1.5 g kg^–1^ day^–1^, which is the amount recommended by the Society for Sarcopenia, Cachexia, and Wasting Disease to maximize muscle health in advanced age (Morley et al., 2010).

Besides quantity, the quality of ingested protein plays an important role in the context of muscle health (Calvani et al., 2013; Landi et al., 2013b). In particular, given the role of leucine as the master dietary regulator of muscle protein turnover, supplementation with protein sources enriched with this essential amino acid is thought to offer the greatest advantage in terms of preservation of muscle mass and function (Paddon-Jones and Rasmussen, 2009; Landi et al., 2013b). This evidence is in line with our finding in that both total protein ingestion and leucine consumption are positively correlated with muscle mass in hip-fractured elderly patients (Figures 2 and 3).

Albeit dealing with a highly relevant issue, our study presents several limitations that need to be discussed. First of all, the study is exploratory in nature, evident by the relatively small sample size. In addition, the cross-sectional design does not allow determining the impact of low protein-energy intake and reduced muscle mass on out-of-hospital survival and functional recovery. Along the same line, the specific impact of individual nutrients on muscle mass could not be established. Although BIA is an established technique for the estimation of lean body mass (Kyle et al., 2003), it does not represent the gold standard for the quantification of muscle mass (Cesari et al., 2012). However, BIA measurements were taken directly at the patient bed, which allowed minimizing discomfort in the pre-operative phase. The lack of a control group of non-hip-fractured older subjects does not allow establishing whether their nutritional habits differ substantially from those of hip-fractured elderly. For the same reason, no information can be provided on eventual differences in the relationship between dietary intake and muscle mass among fractured and non-fractured older adults. Finally, dietary assessment in older adults poses special challenges due to possible memory and cognitive impairment, hearing problems, or biases in diet reporting (Thompson and Subar, 2012). Indeed, because of the high prevalence of chronic illnesses in this age group, it is likely that prescription diets (e.g., low sodium, low fat, and high fiber) are recommended. However, individuals may report what they should eat rather than what they actually eat. Alternatively, subjects on special diets may be more accurate in reporting their actual food consumption. The dietary assessment tool chosen for the present investigation allowed collecting reliable information about food consumption, while avoiding drops in concentration due to excessively long interviews (Adamson et al., 2009). In addition, the exclusion of cognitively impaired patients increased the accuracy and reliability of dietary testing.

## Conclusion

The worldwide epidemic of hip fractures and the dramatic impact on the individual’s health and functionality urge the development of effective strategies for the management of this condition. The recognition of sarcopenia as a major risk factor for adverse outcomes in this patient population indicates that the skeletal muscle may represent a critical target for interventions. The association between low intake of calories, protein and leucine, and reduced muscle mass in hip-fractured older patients revealed by the present study highlights the importance of a comprehensive dietary assessment for the early detection of nutritional deficits, which may aggravate muscle wasting. The evidence provided by this investigation could eventually serve as the foundation for the design of studies testing whether the implementation of nutritional interventions targeting the skeletal muscle (e.g., protein and leucine supplementation) improves the clinical outcomes of older hip-fractured patients.

## Conflict of Interest Statement

The authors declare that the research was conducted in the absence of any commercial or financial relationships that could be construed as a potential conflict of interest.
